# A polymorphic (GA/CT)*n*- SSR influences promoter activity of *Tryptophan decarboxylase* gene in *Catharanthus roseus* L. Don

**DOI:** 10.1038/srep33280

**Published:** 2016-09-13

**Authors:** Santosh Kumar, Sabhyata Bhatia

**Affiliations:** 1 National Institute of Plant Genome Research, Aruna Asaf Ali Marg, PO Box 10531, New Delhi 110067, India

## Abstract

Simple Sequence Repeats (SSRs) of polypurine-polypyrimidine type motifs occur very frequently in the 5′ flanks of genes in plants and have recently been implicated to have a role in regulation of gene expression. In this study, 2 accessions of *Catharanthus roseus* having (CT)_8_ and (CT)_21_ varying motifs in the 5′UTR of *Tryptophan decarboxylase* (*Tdc*) gene, were investigated for its role in regulation of gene expression. Extensive *Tdc* gene expression analysis in the 2 accessions was carried out both at the level of transcription and translation. Transcript abundance was estimated using Northern analysis and qRT-PCR, whereas the rate of *Tdc* gene transcription was assessed using *in-situ* nuclear run-on transcription assay. Translation status of *Tdc* gene was monitored by quantification of polysome associated *Tdc* mRNA using qRT-PCR. These observations were validated through transient expression analysis using the fusion construct [CaM35S:(CT)_8–21_:GUS]. Our study demonstrated that not only does the length of (CT)*n* -SSRs influences the promoter activity, but the presence of SSRs *per se* in the 5′-UTR significantly enhances the level of gene expression. We termed this phenomenon as “microsatellite mediated enhancement” (MME) of gene expression. Results presented here will provide leads for engineering plants with enhanced amounts of medicinally important alkaloids.

Simple sequence repeats (SSRs) or microsatellites occur ubiquitously in eukaryotic genomes as tandem reiterations of short sequence motifs. They exhibit extensive length polymorphisms due to variation in the copy number of repeat motifs and are considered as genetic markers used in DNA fingerprinting, analysis of genetic diversity and linkage mapping. Numerous lines of evidence now suggest that SSRs are non-randomly distributed across transcribed regions of plant genomes^1^,^2^ wherein, UTRs harbor more SSRs than the coding regions^1^,^2^,^3^,^4^,^5^,^6^. Moreover, the 5′-UTRs in particular, contain a majority of di- and tri-nucleotides that exhibit a strong bias towards polypurine-polypyrimidine sequences such as GA/CT and CTT/GAA repeats^4^,^5^,^6^,^7^. Such DNA elements, which till some time ago were termed as “junk DNA,” can have multiple roles in the genomes of higher eukaryotes and have often been found to be associated with gene regulation based on their location in the genome^8^,^9^.

Several studies in the animal kingdom have indicated the functional role of polypurine-polypyrimidine sequences in gene expression through transcription factor binding, methylation of CpG and/or DNA structure modification^2^. In particular, the ‘GAGA’ elements comprising of the dinucleotide repeat sequence (GA)*n*/(CT)*n* have been found in the promoters of numerous genes^10^,^11^,^12^,^13^,^14^,^15^,^16^.

GAGA elements have been most thoroughly examined in *Drosophila*, where they have been shown to be present in the promoter regions and are involved in the regulation of developmental genes by binding to a protein called the GAGA factor, which results in local nucleosome disruption thereby allowing gene expression^17^. A recently described GAGA like element (GLE), homologous to GAGA factor binding sequences, regulate core promoter activity and preferentially affect the transcription start site selection clustered around the 5′ end of core promoter^18^.

However, in plants, such studies are limited. One of the earliest known example in plants is the soybean *Gsa1* that encodes the chlorophyll heme synthesis enzyme Glu2-semialdehyde aminotransferase and has a (GA)_9_/(CT)_*9*_ element in its promoter that has been implicated in regulating expression of that gene in a tissue specific manner^19^,^20^. In another study, a polymorphic (CT)*n* microsatellite identified in the 5′UTR region of the *wx* gene of rice^21^ was correlated with amylose content and microsatellite length polymorphism was thought to affect the expression of the related genes of amylose synthesis^22^. Recently, Joshi-Saha and Reddy^23^ have suggested that (CT)*n* repeat length variation in 5′-UTR of the chickpea *myo-inositol monophosphatase gene* (*CaIMP*), might regulate phytic acid levels to confer drought tolerance in natural populations of chickpea. Additionally, at the whole genome level it has been shown that ~10% of 5′ non-coding CT/GA and CTT/GAA repeats are conserved in *Arabidopsis* and are thought to be involved in regulation of gene expression in plant-specific pathways^6^.

More recently, while analyzing the *Catharanthus roseus* transcriptome we have demonstrated that GA/CT and GAA/CTT repeats were most frequent in 5′-flanks of genes that are known to be involved in enzymatic, regulatory and housekeeping functions^7^. Such preferential distribution and conservation of SSRs in the 5′-UTRs^1^,^7^ strongly suggests that they may be strong contenders for being characterized as a regulatory element. However, more comprehensive analysis needs to be undertaken in order to assess their role in regulating gene expression especially in plant species.

*Catharanthus roseus* is a model medicinal plant species that produces a wide array of pharmaceutically important alkaloids, including anticancer drugs such as vincristine and vinblastine^24^,^25^. These alkaloids are produced in low quantities, making extraction and purification difficult which leads to high market price and poor availability. The terpenoid indole alkaloids originate from tryptophan via the TIA biosynthetic pathway which has been thoroughly investigated^26^,^27^,^28^, but most of it still remains largely unresolved. Tryptophan decarboxylase (TDC) catalyses the first committed step of indole alkaloid synthesis by decarboxylation of tryptophan to form tryptamine^29^ and is a key enzyme in the biosynthetic pathway by virtue of its position at the interface of primary and secondary metabolism. It has been characterized in *C*. *roseus*^28^,^29^,^30^ but its complex, coordinated regulation needs to be investigated in order to exploit it for genetically engineering plants with enhanced levels of useful indole alkaloids.

Therefore, our aim in the present study was to investigate the effect of (CT)*n* microsatellite length variation on *Tdc* gene expression since the *Tdc* genes have been shown to possess variable number of CT motifs in their 5′-UTRs^31^, but the functional role of these SSRs has not been elucidated. It is expected that a thorough investigation of the variations in the number of microsatellite repeat motifs near the TSS within individual accessions of *C*. *roseus* would provide new information with regard to the putative function of these microsatellites. To the best of our knowledge, this is the first time that the functional role of microsatellites (especially CT repeats), present in the 5′UTR of *Tdc* gene of the medicinally important plant *C*. *roseus* is being described. In this study, through parallel measurement of transcript abundance vs rate of transcription and translation, we demonstrate that the plant (CT)*n* repeats indeed modulate the promoter activity of *Tdc* gene in a length dependent manner.

## Results

### Analysis of sequence variation in the 5′-UTR of *Tdc* gene in *C*. *roseus*

**Figure 1:**
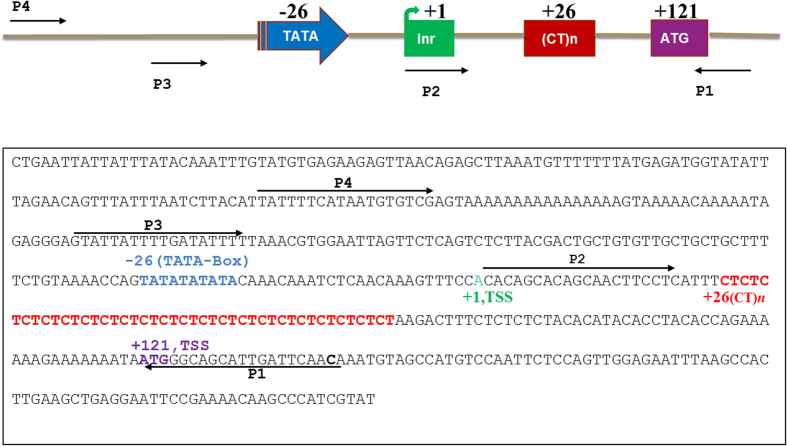
Schematic representation of *C*. *roseus Tdc* basal promoter sequence of approximately 400 bp reproduced from Ouwerkerk *et al.*^33^. TATA-box located at −26, TSS (+1 green), the (CT)*n* repeat motifs at +26 (red) and translation start site at +121(violet) relative to transcription start site. This sequence region was used for designing various primer pairs for subsequent experiments. Primers P1 and P3 (expected size 203 bp) was used for the analysis of microsatellite length polymorphism in *C*. *roseus* accessions. Primers P1 with Xba1 site at 5′ end and P2 with Xma1 site at the 5′ end were used for cloning the (CT)*n* region (expected size of inserts: 129 bp in Kew1 and 155 bp in Prabal) into plant expression vector (pBI121). Primers P1 and P4 (expected size 317 bp) were used for promoter analysis in the two *C*. *roseus* accessions (Kew1 and Prabal).

**Figure 2:**
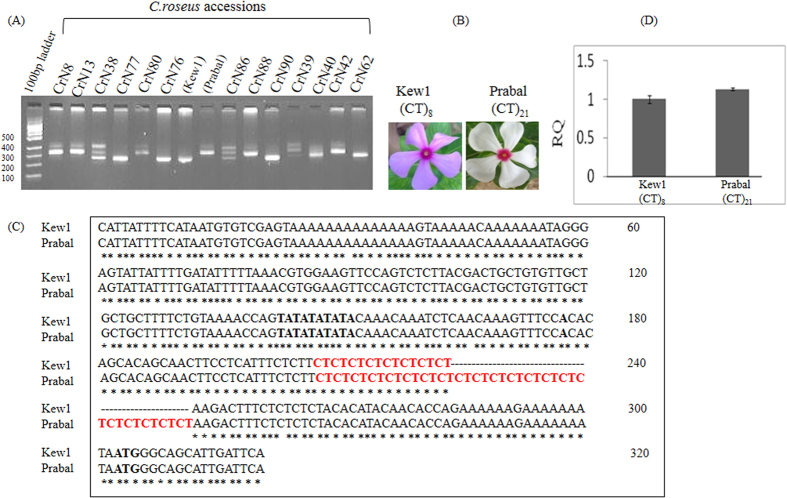
(**A**) PCR amplified 5′UTR regions from 15 *C*. *roseus* accessions were analyzed on Metaphor agarose gel. Length polymorphism across accessions was observed. (**B**) *C*. *roseus* accessions, Kew1, harboring the (CT)_8_ microsatellite motif in the 5′UTR of *Tdc* gene is morphologically distinct from Prabal harboring (CT)_21_ motifs in the *Tdc* gene. Kew1 petals are light pink in colour and central ring is deep pink. However, Prabal petals are white in colour and central ring is deep pink (**C**) CLUSTAL-W sequence alignment of *Tdc* promoter region (approximately 300 bp) of the two *C*. *roseus* accessions, Kew1 and Prabal. Variation in the number of CT motifs was distinctly observed and (**D**) *Tdc* gene copy number variation was analyzed by real time PCR in which genomic DNA of Kew1 and Prabal accessions were used as template for real time amplification of *Tdc* gene. No variation in copy number of *Tdc* was observed.

The *Tdc* gene, which catalyses the conversion of tryptophan to tryptamine in the TIA pathway, had been cloned and characterized from *C*. *roseus*^32^ and shown to possess (CT)*n* repeats in the 5′-UTR region^32^,^33^. Moreover, an earlier study in our lab had shown (CT)*n* polymorphism in the 5′ UTR region of *Tdc* gene^31^. Therefore in the present study, primers P1 and P3 were designed from the regions flanking the (CT)*n* motifs in the 5′UTR of *Tdc* gene (Fig. 1). PCR amplification of genomic DNA from 15 *C*. *roseus* accessions collected from diverse geographical locations was performed using the primers P1 and P3 (Fig. 1). The amplified products revealed bands of various lengths ranging in size from 200 bp to 350 bp in the different accessions (Fig. 2A). The *C*. *roseus* accessions Kew1 and Prabal showing significant length variation were chosen for further studies.

To begin with, the minimal promoter sequences of the *Tdc* genes^33^ of the two *C*. *roseus* accessions, Kew1 and Prabal, were compared. For this, genomic DNA of *C*. *roseus* accessions Kew1 and Prabal was isolated and the *Tdc* 5′UTR as well as the basal promoters were amplified and sequenced using gene specific primers (P1 and P4). Sequence alignment revealed that significant variation existed in the number of (CT)*n* microsatellite motifs in the basal promoter regions of the *Tdc* genes of the 2 *C*. *roseus* accessions, wherein Kew1 had (CT)_8_ motifs, Prabal had (CT)_21_ motifs (Fig. 2C). Sequence comparison also revealed that no other nucleotide variation was present within the basal promoter (~300 bp) region except the variation of the (CT)*n* motif number (Fig. 2C).

Further, to confirm the copy number of *Tdc* gene in the accession Kew1 and Prabal, qPCR was performed. The real time PCR analysis showed that both the accessions of *C*. *roseus* had the same copy number of *Tdc* gene as no significant variation in levels of amplified product (*Tdc* gene) from the genomic DNA was observed [Fig. 2(D)]. Our results demonstrated that the 5′UTRs of the *Tdc* genes of the two accessions Kew1 and Prabal differed only in number of (CT)*n* motifs. No other sequence variation was found in the (CT)*n* flanking regions in the *Tdc* 5′UTR and also the copy number of *Tdc* gene was found to be same in both the accessions, Kew1 and Prabal.

### Estimating the effect of (CT)*n* motif number variation on transcript abundance

**Figure 3:**
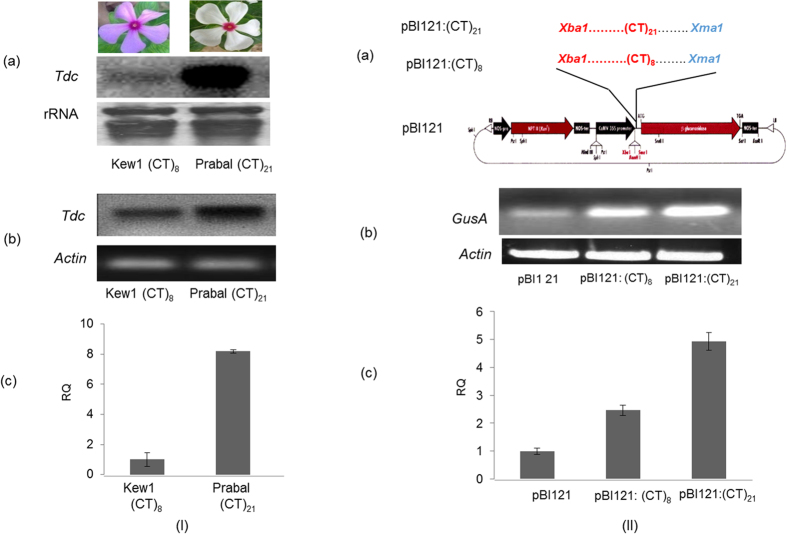
(I) Expression analysis of *Tdc* gene in the two *C*. *roseus* accessions, Kew1 and Prabal having (CT)_8_ and (CT)_21_ motifs respectively in the 5′UTR of *Tdc* gene (**a**) Northern blot analysis. 10 μg of total RNA was probed with cDNA. Ribosomal RNA was used as loading control (**b**) Semi quantitative PCR analysis: endogenous actin gene was used as positive control and (**c**) Real Time quantification of *Tdc* mRNA. It revealed approximately 8 times higher expression in Prabal (CT) _21_ than Kew1 (CT)_8._ (II). Schematic representation of cloning of 5′-UTRs of *Tdc* harboring (CT)_n_ repeat motifs into pBI121 expression vector using *Xba1* and *Xma1* restriction enzymes. The sequences of recombinant constructs were confirmed by sequencing (**a**) Sequence of 5′-UTR (129 bp from Kew1 accession) harboring (CT)_8_ motifs in pBI121:(CT)_8_ construct and 5′-UTR (155 bp from Prabal accession) harboring (CT)_21_ motifs in pBI121:(CT)_21_ construct. Analysis of transiently expressed *GusA* mRNA, driven by respective recombinant pBI121 vectors, by (**b**) semi-quantitative PCR. Tobacco actin gene was used as a control and (**c**) Real time PCR expression analysis of transiently expressed *GusA* gene transcript, wherein pBI121 empty vector was used as a control. Relative expression profile of recombinant vectors pBI121:(CT) _8_ and pBI121:(CT)_21_. (The original figure from which Fig. 3.I (a) was cropped and derived is present as Supplementary Figure 1).

Gene expression analysis is of utmost importance and its measurement may be done efficiently by estimating the mRNA abundance. Therefore, gene expression analysis at the mRNA level was carried out by (1) hybridization based Northern analysis (2) mRNA copy number based semi-Q PCR and (3) real time PCR. Total RNA was isolated from the *C*. *roseus* accessions, Kew1 and Prabal and Northern hybridization analysis was performed using radio labeled *Tdc* cDNA probe. This revealed differential expression of the *Tdc* gene between the two accessions. In Prabal, which had (CT)_21_ di-nucleotide repeat motifs, the *Tdc* gene transcript was more abundant in comparison to Kew1 which had only (CT)_8_ di-nucleotide repeat motfs [Fig. 3I(a)]. Furthermore, quantification of expression was done by semi-Q PCR and real time PCR experiments using cDNA template made from same sample of RNA that was used for Northern analysis. The semi-Q PCR [Fig. 3I(b)] as well as real time PCR [Fig. 3I(c)] also revealed higher levels of expression of *Tdc* gene in Prabal [(CT)_21_] in comparison to Kew1 [(CT)_8_]. Real time data suggested that the *Tdc* transcript abundance in Prabal was approximately 8 times more in comparison to Kew1 [Fig. 3I(c)], thereby validating the results of Northern analysis.

Further, transient expression analysis in *Nicotiana benthamiana* was also carried out. For this, the 5′-UTRs of *Tdc* genes harboring the microsatellite motifs (CT)_8_ and (CT)_21_ were cloned into the pBI121 vector upstream of the reporter gene *gusA* [Fig. 3II(a)] and transformed into *agrobacterium* as described in Methods. The pBI121 empty vector was also transformed into *agrobacterium* and used as a control. The positive recombinant clones were used for agro-injection into tobacco (*N*. *benthamiana*) plants. Leaf discs were harvested from agro-injection sites after three days and total RNA was isolated and subjected to the transient expression analysis of *gusA* gene by using (A) semi-quantitative PCR and (B) real time PCR, using *gusA* specific primers. This revealed differential expression of *gusA* transcripts in tobacco leaves transformed with construct pBI121:(CT)_8_ and pBI121:(CT)_21_ [Fig. 3II(b,c)]. The maximum expression of *gusA* gene was seen in the plant transformed with recombinant pBI121:(CT)_21_, whereas *gusA* expression in plant agro-injected with recombinant pBI121:(CT)_8_ was two-fold less as compared to recombinant pBI121:(CT)_21_ but two-fold excess as compared to control (Fig. 3IIc). The above expression analysis revealed that the (CT)*n* copy number dependent mRNA abundance of *Tdc* gene in *C*. *roseus* was observed both *in situ* and in heterologous reporter gene (*gusA*) expression, thereby establishing that microsatellite length variation in the 5′-UTR of a gene may directly affect transcript abundance.

### Assessing the effect of (CT)*n* length variation on rate of gene transcription and translation

**Figure 4:**
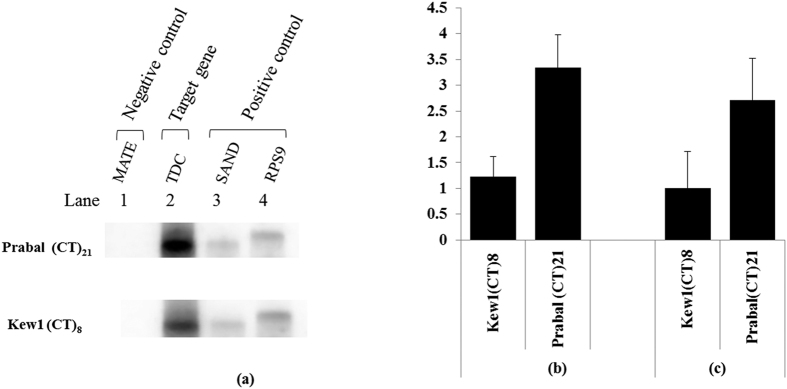
Resolution of flow of information both at the level of transcription and translation. (**a**) Nuclear run-on transcription to measure rate of *Tdc* gene transcription. PCR amplified DNA of *Tdc* (target gene), *Rps9* and SAND (positive control) and MATE (negative control) was blotted on to the nitrocellulose membrane and hybridized with the nascent UTP labeled run-on transcripts from Kew1 and Prabal. (**b**) Quantitative RT-PCR for quantification of *Tdc* mRNA cell extract of Kew1 and Prabal (**c**) Quantitative RT-PCR for quantification of polysome associated *Tdc* mRNA in Kew1 and Prabal. (The original figure from which Fig. 4.(a) was cropped and derived is present as Supplementary Figure 2).

Flow of genetic information follows the path of transcription and translation. However, sometimes mRNA abundance may not be directly indicative of the rate of transcription or translation of a given gene. Therefore to investigate the role of SSRs in transcription and/or translation the following two experiments were performed (A) Nuclear run-on transcription assay to assess the affect of (CT)*n* length variation on rate of transcription and (B) Quantification of polysome associated *Tdc* mRNA to assess the effects of SSRs variation on translation. In nuclear run-on transcription assay the intact nuclei of *C*. *roseus* accessions Kew1 and Prabal was used to synthesize the radio labeled transcripts. These were hybridized with cold probe made of PCR amplified cDNA of *Rps9*, SAND (positive control), *Tdc* (target gene) and MATE (negative control) on membrane. The indicated that the rate of *Tdc* gene transcription was significantly higher in Prabal accession which contained 21 (CT)*n* motifs, as compared to Kew1 having only 8 (CT)*n* motifs [Fig. 4(a)]. This clearly suggested that (CT)*n* length variation contributed in promoter activity of *Tdc* gene.

Further, to assess the role of (CT)*n* motifs in translation, *Tdc* mRNA from cell extract and polysomal fractions from both the *C*. *roseus* accessions, were parallely quantified by quantitative real-time PCR. This revealed no significant variation in expression pattern between steady-state mRNA and transnationally engaged *Tdc* mRNA in accessions Kew1 and Prabal [Fig. 4(b,c)]. The coefficient of correlation between them was found to be significantly high (r = 0.99). This suggested the putative involvement of the (CT)*n* motif number variation in the process of transcription rather than translation.

### *In-vitro* protein-microsatellite interaction

**Figure 5:**
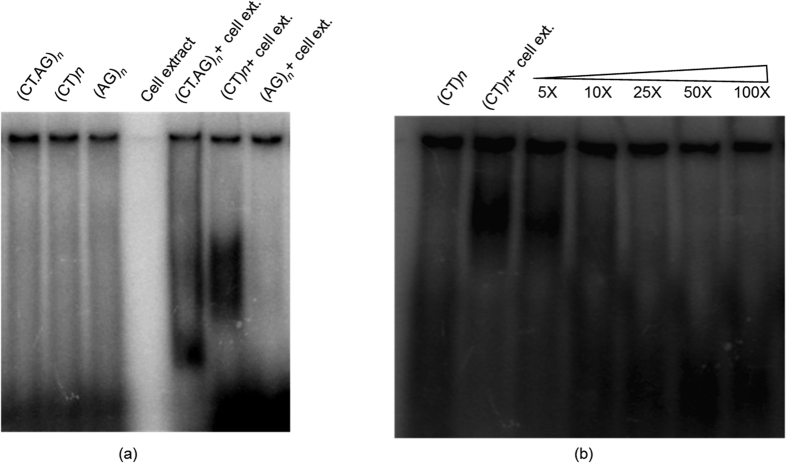
Interaction of protein from cell extract with dinucleotide repeat motifs: EMSA was carried out with three oligonucleotide probes (i) single stranded (CT)_*n*_ (ii) single stranded (GA)_*n*_ and (iii) double-stranded (CT/GA)_*n*_ [*n* = 21]. (**a**) Band-shifting of band was observed in case of single stranded (CT)_21_ with respect to free probe and (**b**) The protein- microsatellite binding affinity was confirmed by using SS(CT)*n* as cold probe (non radioactive), wherein 5X, 10X, 25× 50X, 100X refer to molar excess of cold probe included in the reaction.

**Figure 6:**
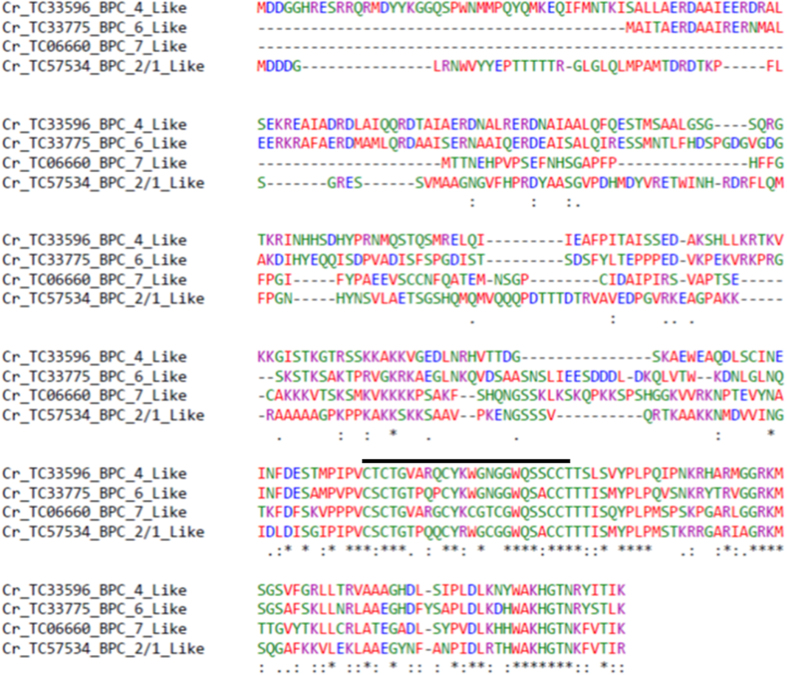
CLUSTAL W alignment of putative BPC/BBR protein from *C*. *roseus*. Putative protein sequences of Cr_TC33596, a BPC_4 like protein, Cr_TC33775, a BPC_6 Like protein, Cr_TC06660, a BPC_7 like protein and TC_57534, a member of BPC_2/1 like proteins were subjected to CLUSTAL W alignment. This revealed all the members of *C*. *roseus* BPCs/BBR have the characteristic conserved pentacysteine C-terminal DNA binding domain.

To investigate the role of the microsatellite sequence as a putative *cis*-acting regulatory element, *in-silico* analysis was performed using the plant *cis*-acting responsive elements (Plant*CARE*) database^34^, The 5′-UTR of *Tdc* gene containing the microsatellite motif (CT)*n* was used as the query. The output result revealed that a protein factor from *Lycopersicon esculentum* recognized the (CT)*n* motif and had a putative role in transcription (Table 1). Therefore, in order to establish the protein-microsatellite interaction, EMSA was performed using the *C*. *roseus* cell extract and three oligonucleotide probes [single stranded (SS) (CT)*n*, (SS) (GA)*n* and double stranded DS (CT/GA)*n*]. Band shifting was observed preferentially with single stranded (CT)*n* [Fig. 5(a)]. However, with other conformations such as SS (GA)*n* and DS (CT/GA)*n*, no protein-DNA interaction was observed. Further, competition assay was performed for confirmation of affinity of (CT)*n*-protein interaction, which showed a gradual fading of band intensity from 5× to 10×, which finally disappeared at 25×, 50×, and 100× of cold probe[Fig. 5(b)]. The present experiment demonstrated that a protein factor, which probably could be a transcription factor, interacted specifically with the single stranded (CT)*n* element. Further, it had been earlier reported that members of a plant specific transcription factors family known as the basic pentacysteine proteins (BPC) or barley B recombinants proteins (BBR), specifically interact with the CT/GA motifs^20^. Therefore, in*-silico* analysis of the available *C*. *roseus* transcriptome^7^ was carried out in order to identify the members of the BPCs/BBR TFs. This resulted in the identification of four members of BPCs/BBR TF family in *C*. *roseus*. These members are Cr_TC33596, a BPC_4 like protein, Cr_TC33775, a BPC_6 like protein, Cr_TC06660, a BPC_7 like protein and TC_57534, a member of BPC_2/1 like protein. CLUSTAL W alignment of these proteins showed that all the members of the *C*. *roseus* BPCs/BBR contained the characteristic conserved pentacysteine C-terminal DNA binding domain (Fig. 6).

## Discussion

This study examines the putative role of (CT)*n*- 5′UTR SSR, as well as its repeat length variation, in transcriptional regulation of *Tdc* gene of model medicinal plant *C*. *roseus*, *in-vivo*. Microsatellite repeats, especially the (CT)*n* motifs, are known to exist in abundance in the 5′UTRs of several eukaryotic genes including plants^5^,^6^,^7^,^35^. However, the involvement of the microsatellite motif *per-se* or based on their length variation, in transcriptional regulation is only beginning to be investigated in plants^23^.

Hence, to elucidate the role of (CT)*n* variation in the 5′UTR of *Tdc* gene of *C*. *roseus*, a systematic approach utilizing (CT)*n* variant alleles from 2 accessions was followed. While selecting the microsatellite for analysis in this study, the following criteria were considered (i) the position/location of microsatellite since regional preference is important as those present in the vicinity of TSS are more likely to be involved in transcription/translation (ii) the variation in the microsatellite repeat motif number - longer repeats were preferred since they have greater probability of being polymorphic and (iii) the nature of repeat motif- the polypurine-polypyrimidine were preferred since they have a higher probability of conformational polymorphism. These criteria were especially important since they increased the likelihood of the chosen microsatellite being a part of the gene regulatory circuits.

Analysis of the sequence variation in the 5′UTR and upstream region of *Tdc* gene from the 2 *C*. *roseus* accessions clearly revealed that no nucleotide variation existed in the regulatory regions of *Tdc* genes of both the accessions, except the variation in the number of polypurine-polypyrimidine (CT)*n* motifs wherein Kew1 possessed (CT)_8_ and Prabal had (CT)_21_ motifs. Further, qRT-PCR was adopted to estimate the relative copy number of *Tdc* gene in the *C*. *roseus* accessions. The copy number of a gene in general is determined by Southern blotting^36^. However, recently quantitative RT-PCR assay has been successfully used to determine gene copy number^37^,^38^. In this study, quantitative real- time PCR analysis revealed no relative difference in the *Tdc* gene copy number in the 2 *C*. *roseus* accessions, Kew1 and Prabal since no significant variation in the level of *Tdc* amplification was observed. Since the findings of an earlier report^32^ had suggested that in *C*. *roseus*, TDC was encoded by an intron less single copy genetherefore taken together it may be suggested that both the *C*. *roseus* accessions had single copy of *Tdc* gene.

The untranslated mRNA leader sequences (UTRs) or downstream promoter sequences (DPE) are thought to play a pivotal role in the regulation of eukaryotic gene expression at various levels: transcriptional, post-transcriptional and translational^23^,^39^,^40^,^41^,^42^,^43^,^44^. Moreover, presence of SSRs in the leader sequence or within promoter regions are being implicated in regulation of gene expression. For example, the (CTC)*n* microsatellites located in the promoter of the paired box gene 7 (*PAX7*) was found to have three different alleles, with 8, 10, or 11 repeats and it was found that the alleles with 11 repeats gave a significantly higher transcriptional activity than alleles with 8 or 10 repeats^45^. In rice tungro bacilliform virus (RTBV), the DNA sequence from +50 to +90 stimulates RTBV promoter activity in a copy number dependent manner^46^.

In soybean *Gsa1* that encodes the chlorophyll heme synthesis enzyme Glu2-semialdehyde aminotransferase and has a (GA)_9_/(CT)_*9*_ element in its promoter that has been implicated in regulating expression of that gene in a tissue specific manner^19^,^20^. In another study, a polymorphic (CT)*n* microsatellite identified in the 5′UTR region of the *wx* gene of rice^21^ was correlated with amylose content and microsatellite length polymorphism was thought to affect the expression of the genes related to amylose synthesis^22^. More recently it was shown that the length variation of (CT)*n* repeat motifs present in the 5′UTR of *CaIMP* leads to differential transcription in chickpea drought tolerant and susceptible accessions. The *CaIMP* allele with the smaller number of (CT)_9_ repeat motifs in drought tolerant accession was almost 2-fold up regulated as compared to a susceptible accession having the longer (CT)_25_ repeat^23^.

Similarly in our study, comparison of *Tdc* gene expression in the 2 *C*. *roseus* accessions varying in the (CT)*n* motif numbers [(CT)_8_ and (CT)_21_] revealed that motif number variation in the regulatory non-coding element (5′UTR) of *Tdc* gene may be responsible for significant quantitative and temporal difference in gene expression between the *C*. *roseus* accessions. This was clearly established by measuring *Tdc* mRNA abundance through Northern analysis and qRT-PCR analysis, where the accession with longer repeats (CT)_21_ showed higher abundance of *Tdc* mRNA transcript than accession with shorter repeats (CT)_8_.

Our results also demonstrated that the (CT)*n* repeat motifs from +24 to +66 stimulate *Tdc* promoter activity in a copy number dependent manner. Transient expression analysis of plants transformed with 5′-UTR of *Tdc* harboring two different lengths of repeat motifs [(CT)_8_ and (CT)_21_] cloned upstream of *gus A* reporter gene clearly indicated that the level of expression was dependent on copy number of repeat motif: longer the repeat motif, higher the expression level of reporter gene. This was in agreement with the report of De Amicis *et al*.^47^ where *gusA* leader when replaced with (CT)*n* repeat sequence led to enhanced expression of reporter gene. Hence, the (CT)*n* microsatellite motifs *per se* as well as their copy number variation govern mRNA transcript abundance *in situ* of *Tdc* gene in *C*. *roseus*.

It is well established that transcript abundance has no direct quantitative relationship to its rate of synthesis^43^,^44^. In many cases, certain transcripts maybe are rarely transcribed but may exhibit a prolonged half-life in response to external or internal cues^48^,^49^. In either case, the transcript levels are not true indicators of active transcription and therefore an accurate assessment of transcription rate is often desired to describe the important metrics of promoter activity and relative transcript stability.

Therefore, in this study nuclear run on transcription assay for measurement of the relative rate of *Tdc* gene transcription in *C*. *roseus* was successfully performed. This indicated that the differential mRNA abundance as observed in previous experiment was due to differential rate of transcription of *Tdc* gene in the two *C*. *roseus* accessions. Microsatellite motif number variation mainly affected the synthesis of nascent transcripts rather than transcript stabilization or degradation thereby clearly indicating that the motif number dependent *Tdc* expression was solely responsible for increased promoter activity.

In eukaryotes, the translation efficiency is known to be affected by both leader sequence and structure^40^,^47^,^50^,^51^. In this study, through parallel measurement of transcript abundance and protein synthesis, it was demonstrated that 5′UTR microsatellite length polymorphism [(CT)*n*] was not involved in the process of translation of T*dc* gene. This could be suggested since the pattern of translationally engaged *Tdc* transcripts profile in the 2 *C*. *roseus* accessions were found to be similar to that in transcription, thereby indicating its involvement in regulating transcription rather than translation.

Many recent studies have demonstrated that repeats motifs bind to specific protein factors, which in many cases have been characterized to be TFs. In plants, GAGA binding proteins (GBPs) have been identified and characterized to be plant specific transcription factors, the BASIC PENTACYSTEINE/BARLEY B RECOMBINANT (BPC/BBR). Members of this family are characterized by the ability to bind the DNA at GA- rich sequences. These were originally detected in soybean where GBP binds to the promoter of the heme and chlorophyll synthesis gene *Gsa1*, which contains a GAGA element^20^, and subsequently have been identified in various plant species, such as barley^52^ and Arabidopsis^53^. Interaction of BPC/GBP with CT/GA repeat motifs leads to transcriptional activation or repression of genes^20^,^53^,^54^,^55^,^56^,^57^ involved in a diverse range of developmental processes. For example in barley, the BBR factor directly regulates transcription of the HOMEOBOX transcription factor BKN3^52^. In seed development, BPC acts as a regulator of the B3-domain LEAFY COTYLEDON 2 (LEC2) gene^54^. BPCs are also known to regulate the expression of INNER NO OUTER (INO), a gene involved in ovule development^53^. BPCs also regulate the expression of the ovule identity determinig SEEDSTICK (STK) gene by looping its regulatory region and through interaction with a MADS domain transcription factor containing repressor complex^56^.

The EMSA analysis carried out in our study also revealed that a protein factor exclusively interacts with the single strand conformation of (CT) motifs but not with SS (GA) motif or double stranded CT/GA motifs. Further, through *in-silico* analysis of the *C*. *roseus* transcriptome^7^,^58^, four members of BPC/BBR TF were identified that had conserved pentacysteine C-terminal DNA binding domains (Fig. 6). Taken together, our results suggested that the CT repeat motifs might be a specific *cis* element with which a protein, most likely a member of BPC/BBR transcription factor family, may interact for regulating *Tdc* gene transcription *in-vivo*.

Earlier studies have suggested that the SSRs may act as “tuning knobs”^59^,^60^ for modulation of gene expression, wherein the larger the number of the repeats in tandem, finer the tuning. The changes in the “tuners” of relevant genes in desirable directions and respective changes of the gene activities towards mitigation of the stress therefore get selected^60^. *Tdc* is an important gene of the alkaloid producing pathway (TIA) in *C*. *roseus* and is known to be induced under several biotic and abiotic stresses. In this context our findings therefore valuable for genetic engineering of TIA pathway in *C*. *roseus* for enhanced production of alkaloid. In summary, this study presents an exhaustive investigation of the putative role of 5′UTR-SSRs in regulation of gene expression. Our study suggests that the (CT)*n* microsatellite sequence flanking the transcription start site might be a new *cis-*acting, down-stream promoter element (DPE) or enhancer element. Binding of proteins at tandemly repeated (CT)*n* sequences may influence *Tdc* gene transcription in a length dependent fashion. We termed this phenomenon as **“microsatellite mediated enhancement”** (**MME**) of gene expression. The results presented here will serve as a foundation for further elucidating the mechanism of 5′UTR SSRs mediated gene expression and also provide leads for engineering plants with enhanced amounts of medicinally important indole alkaloids.

## Materials and Methods

### Biological materials

Fifteen *C*. *roseus* accessions (Listed in Fig. 2A), were grown in the field and under controlled conditions in the phytotron facility (at NIPGR) as per requirement of experiments. *C*. *roseus* accessions (Kew1 and Prabal) were also grown in greenhouses with a 16-h (natural daylight) photoperiod and a 25/22 °C day-night temperature regime. Tissue samples were harvested in triplicates from the 2 accessions at same stage of development i.e. four week old seedlings. *Nicotiana benthamiana* plants were grown in green house at 22 °C for 6 weeks. For transformation, we used *agrobacterium tumefaciens* strain GV3101 and plant expression vector pBI121. Sample infiltrated leaf were harvested in triplicate with each leaf having 8–16 infected spots with sizes of typically 3–4 cm^2^ in infiltrated area separated by veins on a single leaf.

### Analysis of allelic polymorphism, promoter sequence and gene copy number

Genomic DNA was isolated from leaf samples of *C*. *roseus* accessions using GenElute^TM^ Plant Genomic DNA Miniprep Kit (SIGMA). Quantity and quality was checked on the Nano-drop instrument (Thermo Scientific). PCR amplification of genomic DNA was performed with gene specific primers [P1: 5′-TGAATCAATGCTGCCCATTAC and P3: CTGTGTTGCTGCTGCTTTTC (Fig. 1)]. The PCR reaction mixture contained 50 ng DNA, 1.0 μl of 10 μM of each primer, 1.0 μl of 10 mM dNTPs, 2.0 ul of 10X buffer and 1.0 μl of Titanium Taq DNA polymerase. Reaction conditions were as follows: initial denaturation at 95 °C for 2 min, followed by 35 cycles of denaturation at 95 °C for 20 sec, annealing at 60 °C for 30 sec and extension at 72 °C for 1 min with final extension at 72 °C for 10 min. The PCR products were electrophoresed on 3% metaphor agarose gel at 6 V/cm for 2 h in 1XTBE. The polymorphic bands were eluted using HiYield^TM^ Gel/PCR Mini Kit (RBC) followed by cloning into pGEM-T easy vector (Promega). The putative clones were confirmed by sequencing using Big Dye Terminator kit version 3.0 (ABI, USA) and analyzed with the 3700 ABI Prism 96 capillary sequence analyzer followed by sequence comparison using CLUSTAL-W program of NCBI. For promoter sequence analysis of *Tdc* genes from 2 *C*. *roseus* accessions, genomic DNA was amplified using primers pair [P1: TGAATCAATGCTGCCCATTAC and P4 CATTATTTTCATAATGTGTCGA (Fig. 1)] flanking the minimal *Tdc* promoter sequence. The PCR amplified product were then cloned, sequenced and pair wise alignment was made.

To assess the gene copy number, Quantitative RT PCR^61^ was performed on an Applied Biosystem Step One^TM^ Real Time PCR system using 10 ng genomic DNA as template, which was added to 18 μl of reaction mixture containing 6.2 μl of sterile MQ-water, 10 μl of Power SYBR green PCR master mix (Applied Biosystems), 0.9 μl (20 pico moles) of each of forward and reverse primers. PCR amplification was carried out for 50 cycles of 94 °C for 10 sec, 76 °C for 15 sec and 72 °C for 55 sec. The temperature range for the analysis of melting curve was 60 °C to 90 °C over 30 sec. All reactions were performed in triplicate.

### Northern analysis, semi-quantitative and quantitative RT-PCR analysis

For Northern analysis, total RNA was isolated using LiCl method and transferred on to nylon membrane according to Sambrook *et al*.^62^. Probe sample was prepared by random labeling using NEBlot^®^ kit (Amersham Biosciences) and purified by gel filtration on Sephadex G-50 (GE Healthcare) spin column. The purified probe was denatured and used for hybridization for 14–16 hr at 60 °C. Membrane was washed twice with 2X SSC and 0.1% SDS (w/v) followed by autoradiography at −80 °C for 24–48 hrs depending upon the signal intensity.

For the quantification of gene expression, first-strand cDNA synthesis was carried out by using Clontech kit as per manufacturer instructions. The first strand cDNA sample was used as template for semi qRT-PCR analysis, which was carried out in 50 μl of PCR reaction mix having 5 μl of 10X PCR buffer, 4 μl of 2.5 μM dNTP (New England Biolab), 0.2 μl of Titanium Taq polymerase (Clontech) and 1 μl of each gene specific primer. All PCR amplifications (in triplicate) were carried out using the following program: denaturation at 95 °C for 2 min, 21/23/25 cycles at 95 °C for 15 s, 60 °C for 15 s, 68 °C for 1 min and final extension at 72 °C for 7 min. Aliquots were collected after 21 cycles, 23 cycles and 25 cycles of PCR run followed by electrophoresis on 1.2% agarose/EtBr gels.

For real time PCR analysis, the following primers were used to amplify the *Tdc* gene transcript [sense 5′-AGCGAAGTCGAACCTGGATATC and antisense 5′-GGGAGGTAAGGAGCGGTTTC] and the *C*. *roseus* actin gene was amplified using [sense 5′-CTATGTTCCCAGGTATTGCAGATA and antisense 5′-GCTGCTTGGAGCCAAAGC]. Reaction mix was prepared as described above for quantitative RT-PCR. The PCR amplifications consisted of an initial incubation of 2 min at 50 °C followed by denaturation at 95 °C for 10 min, 40 cycles of amplification of 15 seconds at 95 °C and 1 min at 60 °C. Finally, a melt curve analysis was performed from 60 °C to 95 °C in increments of 0.5 °C to confirm the presence of a single product and absence of primer-dimers. Each sample was assayed in triplicates, and each experiment was repeated at least twice. Expression levels were calculated relative to the constitutively expressed gene (actin). Normalization was carried out using the ^∆∆^Ct method (Applied Biosystems), where ∆Ct was calculated for each sample as the difference between Ct of the target gene and Ct of actin, and final relative expression level was determined as inverse of log2 of Ct (target gene) −Ct (actin).

### Transient expression analysis

#### Plasmid construction

For the heterologus gene expression analysis, the 5′ UTR regions of *Tdc* gene from the two *C*. *roseus* accessions Kew1 and Prabal were amplified with UTR specific primer pairs which were designed in such a manner that 5′ end of forward primer had *Xba1* restriction site whereas the 5′ end of reverse primer had *Xma1* restriction site (F: 5′-GCTCTAGAACAGCACAGCAACTTCCTCA and R: 5′-CCCCCGGGTGTGAATCAATGCTGCCCATTA). The PCR amplicon and the pBI121 plant expression vector were double digested with respective enzymes (NEB) followed by purification and ligation using T4 DNA ligase (Clontech). Recombinant and control pBI121 vector was transformed into competent *E*. *coli* cells (NEB-10 beta) and confirmed by sequencing using CaM35S forward primer and *GusA* reverse primers. Further, plasmid DNA was transformed into the competent GV3101 *agrobacterium* strain and selected on media containing 50 μg/ml kanamycin and 30 μg/ml rifampicin.

#### Agro infiltration

The colonies harboring individual constructs [pBI121, pBI121:(CT)_8_ and pBI121:(CT)_21_] were streaked on YEP solid medium (10 g^−1^ bacto-peptone, 10 g^−1^ yeast extract, 5 g^−1^ NaCl, 15 g^−1^ agar) supplemented with rifampicin (60 μg ml^−1^) and kanamycin (50 μg ml^−1^) and grown at 28 °C for 2 days. *Agrobacterium* colonies were then inoculated in 20 ml induction medium containing AB salts (1 g^−1^ NH_4_Cl, 0.3g^−l^ MgSO_4_-7H_2_O, 0.15 g^−1^ KCl, 0.01 g^−1^ CaCl_2_, 0.0025 g^−1^ FeSO_4_-7H_2_O), 2 mM phosphate, 1% glucose, 20 mM 2-(*N-*morpholino) ethanesulfonic acid (MES, pH 5.5), 100 μM acetosyringone as well as rifampicin and kanamycin. After overnight culture at 28 °C, *agrobacterium* cells were pelleted by centrifugation for 15 min at 3000 × g and resuspended in 10 mM MES (pH 5.5) plus 10 mM MgSO_4_ solution plus MS basal medium. After supplementing with 100 μM acetosyringone, bacterial suspension was adjusted to a final OD_600_ of 0.8 for agroinfiltration.

Transient transformation was conducted on fully expanded tobacco leaves that were still attached to the intact plant. Bacterial suspension was infiltrated into intercellular spaces of intact leaves using a plastic syringe of 1 ml. 100 μl of bacterial suspension was infiltrated into 8–16 spots (typically 3–4 cm^2^ in infiltrated area) separated by veins on a single leaf. Leaf discs from each infiltration site were collected and total RNA was isolated for first strand cDNA synthesis as described earlier. Next, the transient expression analysis was performed by semi-quantitative and quantitative PCR as described earlier, wherein, reporter gene *gusA* transcript was amplified by gene specific primer pairs [sense 5′-TGCATCAGCCGATTATCATCA, antisense 5′-GTGCAGCCCGGCTAACG] and control gene *actin* (tobacco) transcripts was amplified by gene specific primer pairs [sense 5′-TGGCATCATACTTTCTACAATGA, antisense 5′-CCCCTCATAAATTGGGACAG].

### Nuclear run-on transcription assay

#### Nuclei isolation and *in-vitro* transcription

Intact nuclei isolation and run-on transcription assay were carried out using the procedure of Kumar and Bhatia^63^. In brief, 5 g of *C*. *roseus* leaf tissue was homogenized and then suspended in 1X extraction buffer (2 M hexylene glycol (2-methyl-2, 4-pentandiol), 20 mM PIPES-KOH (pH 7.0), 10 mM MgCl_2_ and 5 mM 2-mercaptoethanol). The homogenate was filtered and 10% triton-X-100 was added to the solution until the final concentration was 1%. The extract was gently layered onto the top of the Percoll gradient of 30% and 80% assembled in a 50 ml culture tube followed by centrifugation at 2,000 × g for 30 min at 4 °C. The nuclei at the 30–80% interface were collected and washed followed by centrifugation at 900 × g for 5 min. The nuclei pellet was then dissolved in 500 μl of nuclei storage buffer (50 mM Tris–HCl (pH 7.8), 10 mM 2-mercaptoethanol, 20% glycerol, 5 mM MgCl_2_ and 0.44 M sucrose). The run-on reaction (100 μl) was set up using 10 μl of 10X transcription assay buffer [250 mM Tris–HCl (pH 7.8), 375 mM NH_4_Cl, 50 mM MgCl_2_ and 50% (v/v) glycerol], 5 μl of each 100 mM CTP, GTP and ATP, 10 μl of ^32^P-UTP, 50 μl nuclei in storage buffer and 15 μl of water. Reaction was allowed to proceed at 30 °C for 20 min followed by addition of 10 U of DNase I to terminate the reaction. Transcripts were purified and precipitated with 1/10 volume of 7.5 M ammonium acetate (pH 7.0), 100 μg of yeast tRNA along with ice-cold ethanol. Precipitated sample was dissolved in hybridization buffer.

#### Hybridization and autoradiography

The radio labelled run-on transcripts in hybridization buffer (Miracle-Hyb buffer, Stratagene USA) were denatured and hybridized with blots (harboring target (*Tdc*), reference gene (*Rps9*, SAND) and negative control (MATE) for 16 h at 60 °C. The filters were washed three times in 2×SSC, 0.1% SDS at 65 °C, twice in 0.2×SSC, 0.1% SDS at 65C and once in 0.1% SSC, 0.1% SDS at 65 °C. The semi-dried membrane was exposed to hyper screen (Amersham) in a cassette and was kept at room temperature for 1–12 h. Autoradiography was carried out using Molecular Dynamics Phosphor Imager and filter was scanned with high resolution scanner (Typhoon, Amersham USA). The band intensities were quantified with Alpha Imager software (Innotech Corporation, San Lecandro, CA, USA).

### Polysome isolation and quantification of translating *Tdc* mRNA

For polysome isolation from leaf tissue, the modified protocols of Juntawong *et al*.^64^ were used. *C*.*roseus* leaves (0.4 g fresh weight) were homogenized in liquid nitrogen with mortar and pestle and suspended in 2 mL of ribosome extraction buffer (50 mM KCl, 200 mM Tris-acetate, pH 8.5, 25 mM MgCl_2_, 5 mM EGTA, 10 mM 2- mercaptoethanol, 2% polyoxyethylene [10] tridecyl ether, 1% Triton X-100, 100 μg/mL Heparin, 0.5% [v/v] Nonidet P-40, 100 μg/ml chloramphenicol, and 100 μg/ml cycloheximide). The suspension was centrifuged for 10 min at 15,000 g and 4 °C to remove cell debris. A 500 μL aliquot was removed and frozen for subsequent total RNA isolation, cDNA preparation and quantification by qRT-PCR. Aliquots of the resultant supernatant were loaded on 10 to 50% (w/w) continuous sucrose gradients and centrifuged at 38,000 rpm for 3 h in a Beckman 90Ti rotor at 4 °C. After centrifugation, fractions were collected from the bottom to the top of the gradient with continuous monitoring of 254-nm absorbance and the fraction representing polysomes were further subjected to RNA extraction, cDNA preparation and quantification by qRT-PCR.

### Electrophoretic mobility shift assay (EMSA)

The cell extract was prepared using the modified protocol described in CelLytic^TM^ PN plant nuclei isolation/extraction Kit (Sigma) protocol. For preparing the whole cell extract, leaf sample was homogenized and suspended in extraction buffer (20 mM HEPES, 150 mM NaCl, 1 mM EDTA, 1% Triton X-100 and 1:100(v/v) protease inhibitors cocktail), and kept on ice for 30 min followed by centrifugation for 3 min at 200×g. The resulting supernatant was desalted over PD-10 column (Sigma-Aldich) against gravity. Three sets of oligonucleotide probes namely single stranded (CT)_21_, single stranded (GA)_21_, and double stranded (CT/GA)_21_ oligo (annealed) were generated by labelling 100 pmol of oligonucleotide with 5 μl of radio-labeled γ32p-ATP (6,000 ci/mmol, 50 μCi) and 1 μl of T4 polynucleotide kinase (10 unit) for 30 min at 37 °C. Reaction was terminated by heating at 85 °C for 5 min followed by purification by gel filtration on Sephadex G-25 spin column. Labeled DNA was denatured by heating in boiling water bath at 95–100 °C for 5 min. Sample was quickly placed in ice bath for 5 min. The protein-DNA binding reaction was performed in 50 μl reaction volume that consisted of 30 μg cell extract, 1X Binding buffer (20 mM HEPES (pH 7.5), 0.5 mM EDTA, 1 mM MgCl_2,_ 20 mM KCl, 6% glycerol, 1 μg poly (dI-dC) and 0.5 mM DTT) and 50–100 pmol of labeled oligo-nucleotide probe, at room temperature for 20–30 min. the bound products were separated on 6% native polyacrylamide (acrylamide: bisacrylamide, 19:1) gel in 1× TBE at room temperature for 1 h and exposed to autoradiography.

## Additional Information

**How to cite this article**: Kumar, S. and Bhatia, S. A polymorphic (GA/CT)*n*- SSR influences promoter activity of *Tryptophan decarboxylase* gene in *Catharanthus roseus* L. Don. *Sci. Rep.* **6**, 33280; doi: 10.1038/srep33280 (2016).

## Supplementary Material

Supplementary Information

## Figures and Tables

**Table 1:** *In-silico* identification of putative protein factors binding to the promoter of *Tdc* by Plant*CARE*.

Site Name	Organism	Position	Strand	Matrix score.	Sequence	Function	Reference
5UTR Py-rich stretch	*Lycopersicon esculentum*	154	+	14	TTTCTCTCTCTCTC	cis-acting element conferring high transcription levels	34

## References

[b1] Fujimori S. . A novel feature of microsatellites in plants, a distribution gradient along the direction of transcription. FEBS Lett. 554, 17–22 (2003). 14596907 10.1016/s0014-5793(03)01041-x

[b2] Li Y.–C., Abraham, Korol B., Fahima I. & Nevo E. Microsatellites within Genes, Structure, Function, and Evolution. Mol. Biol. Evol. 26, 991–1007 (2004). 14963101 10.1093/molbev/msh073

[b3] Wren J. D. . Repeat polymorphisms within gene regions, phenotypic and evolutionary implications. Am. J. Hum. Genet. 67, 345–356 (2000). 10889045 10.1086/303013 PMC1287183

[b4] Morgante M., Hanafey M. & Powell W. Microsatellites are preferentially associated with nonrepetitive DNA in plant genomes. Nat. Genet. 30, 194–200 (2002). 11799393 10.1038/ng822

[b5] Zhang L. D. . Preference of simple sequence repeats in coding and non–coding regions of *Arabidopsis thaliana*. Bioinformatics. 20, 1081–1086 (2004). 14764542 10.1093/bioinformatics/bth043

[b6] Zhang L. . Conservation of noncoding microsatellites in plants, implication for gene regulation. BMC Genomics. 7, 323 (2006). 17187690 10.1186/1471-2164-7-323 PMC1781443

[b7] Kumar S., Shah N., Garg V. & Bhatia S. Large scale *in-silico* identification and characterization of simple sequence repeats SSRs from de novo assembled transcriptome of *Catharanthus roseus* L. G. Don. Plant Cell Rep. 336, 905–918 (2014). 24482265 10.1007/s00299-014-1569-8

[b8] Castillo-Davis C. I. The evolution of noncoding DNA, how much junk, how much func? Trends Genet. 21(10), 533–536 (2005). 16098630 10.1016/j.tig.2005.08.001

[b9] Simpson P. & Ayyar S. Evolution of cis-regulatory sequences in Drosophila. Adva. Genet. 61, 67–106 (2008). 10.1016/S0065-2660(07)00003-X 18282503

[b10] Gilmour D. S., Thomas G. H. & Elgin S. C. R. Drosophila nuclear proteins bind to regions of alternating C and T residues in gene promoters. Science. 245, 1487–1490 (1989). 2781290 10.1126/science.2781290

[b11] Kerrigan L. A., Croston G. E., Lira L. M. & Kadonaga J. T. Sequence–specific transcriptional antirepression of the Drosophila Kruppel gene by the GAGA factor. J. Biol. Chem. 266, 574–582 (1991). 1985916

[b12] Li J., Liang V. C., Sedgwick T., Wong J. & Shi Y. B. Unique organization and involvement of GAGA factors in transcriptional regulation of the Xenopus stromelysin–3 gene. Nucleic Acids Res. 26, 3018–3025 (1998). 9611250 10.1093/nar/26.12.3018 PMC147655

[b13] Bevilacqua A., Fiorenza M. T. & Mangia F. A. Developmentally regulated GAGA box–binding factor and Sp1 are required for transcription of the hsp70.1 gene at the onset of mouse zygotic genome activation. Development 127, 1541–1551 (2000). 10704399 10.1242/dev.127.7.1541

[b14] Wyse B. D., Linas S. L. & Thekkumkara T. J. Functional role of a novel cis–acting element GAGA box in human type–1 angiotensin II receptor gene transcription. J. Mol. Endocr. 25, 97–108 (2000). 10.1677/jme.0.0250097 10915222

[b15] Busturia A. . The MCP silencer of the Drosophila Abd–B gene requires both pleiohomeotic and GAGA factor for the maintenance of repression. Development 128, 2163–2173 (2001). 11493537 10.1242/dev.128.11.2163

[b16] Hodgson J. W., Argiropoulos B. & Brock H. W. Site–specific recognition of a 70–base–pair element containing dGAn repeats mediates bithoraxoid polycomb group response element–dependent silencing. Mo. Cell Biol. 21, 4528–4543 (2001). 10.1128/MCB.21.14.4528-4543.2001 PMC87112 11416132

[b17] Tsukiyama T., Becker P. B. & Wu C. ATP–dependent nucleosome disruption at a heat–shock promoter mediated by binding of GAGA transcription factor. Nature. 367, 525–532 (1994). 8107823 10.1038/367525a0

[b18] Lee N. . Three Novel Downstream Promoter Elements Regulate MHC Class I Promoter Activity in Mammalian Cells. PLoS One. 512, e15278 (2010). 21179443 10.1371/journal.pone.0015278 PMC3001478

[b19] Sangwan I. & O’Brian M. R. Expression of the soybean (*Glycine max*) glutamate 1-semialdehyde aminotransferase gene in symbiotic root nodules. Plant Physiol. 102, 829–834 (1993). 8278535 10.1104/pp.102.3.829 PMC158853

[b20] Sangwan I. & O’Brian M. R. Identification of a soybean protein that interacts with GAGA element dinucleotide repeat DNA. Plant Physiol. 129, 1788–1794 (2002). 12177492 10.1104/pp.002618 PMC166767

[b21] Blight H. F. J., Till R. I. & Jones C. A. A microsatellite sequence closely linked to the waxy gene of Oriza sativa. Euphytica. 86, 83–85 (1995).

[b22] Bao S., Corke H. & Sun M. Microsatellites in starch–synthesizing genes in relation to starch physicochemical properties in waxy rice *Oryza sativa* L. Theor. Appl. Genet. 105, 898–905 (2002). 12582915 10.1007/s00122-002-1049-3

[b23] Joshi-Saha A. & Reddy K. S. Repeat length variation in the 5′UTR of myo-inositol monophosphatase gene is related to phytic acid content and contributes to drought tolerance in chickpea *Cicer arietinum* L. J. Exp. Bot. 6619, 5683–5690 (2015). 25888598 10.1093/jxb/erv156

[b24] Van der Heijden R., Jacobs D. I., Snoeijer W., Hallard D. & Verpoorte V. The *Catharanthus* alkaloids, pharmacognosy and biotechnology. Curr. Med. Chem. 11, 607–628 (2004). 15032608 10.2174/0929867043455846

[b25] O’Connor S. E. & Maresh J. J. Chemistry and biology of monoterpene indole alkaloid biosynthesis. Nat. Prod. Rep. 23, 532–547 (2006). 16874388 10.1039/b512615k

[b26] Oudin A. . Spatial distribution and hormonal regulation of gene products from methyl erythritol phosphate and monoterpene-secoiridoid pathways in *Catharanthus roseus*. Plant Mol. Biol. 65, 13–30 (2007). 17611800 10.1007/s11103-007-9190-7

[b27] Loyola-Vargas V. M., Galaz-A valos R. M. & Ku-Cauich R. *Catharanthus* biosynthetic enzymes: the road ahead. Phytochem. Rev. 6, 307–339 (2007).

[b28] Miettinen K. . The seco-iridoid pathway from *Catharanthus roseus*. Nature Comms., 10.1038/ncomms4606 (2014). PMC3992524 24710322

[b29] Glenn W. S., Nims E. & O’Connor S. E. Reengineering a tryptophan halogenase to preferentially chlorinate a direct alkaloid precursor. J. Am. Chem. Soc. 133, 19346–19349 (2011). 22050348 10.1021/ja2089348

[b30] Kellner F. . Genome-guided investigation of plant natural product biosynthesis. The Plant J. 82, 680–692 (2015). 25759247 10.1111/tpj.12827

[b31] Shooken B., Chaudhary S., Sethy N. K. & Bhatia S. Development of SSR and gene-targeted markers for construction of a framework linkage map of *Catharanthus roseus*. Annals of Bot. 108, 321–336 (2011). 10.1093/aob/mcr162 PMC3143056 21788377

[b32] Goddijn O. J., Lohman F. P., de Kam R. J., Schilperoort R. A. & Hoge J. H. Nucleotide sequence of the tryptophan decarboxylase gene of *Catharanthus roseus* and expression of tdc-gusA gene fusions in *Nicotiana tabacum*. Mol. Genet. Genom. 2422, 217–225 (1994). 10.1007/BF00391016 8159173

[b33] Ouwerkerk P. B., Hallard D., Verpoorte R. & Memelink J. Identification of UV-B light-responsive regions in the promoter of the tryptophan decarboxylase gene from *Catharanthus roseus*. Plant Mol. Biol. 41(4), 491–503 (1999). 10608659 10.1023/a:1006321100550

[b34] Lescot M. . *PlantCARE*, a database of plant cis-acting regulatory elements and a portal to tools for *in silico* analysis of promoter sequences. Nucleic Acids Res. 301, 325–327 (2002). 11752327 10.1093/nar/30.1.325 PMC99092

[b35] Zhao Z. . Genome- Wide Analysis of Tandem Repeats in Plants and Green Algae. G3: Genes, Genomes, Genetics 41, 67–78 (2014). 24192840 10.1534/g3.113.008524 PMC3887541

[b36] Southern E. M. Detection of specific sequences among DNA fragments separated by gel electrophoresis. J. Mol. Biol. 98, 503–517 (1975). 1195397 10.1016/s0022-2836(75)80083-0

[b37] Schmidt M. A. & Parrott W. A. Quantitative detection of transgenes in soybean [Glycine max L. Merrill] and peanut *Arachis hypogaea* L. by real–time polymerase chain reaction. Plant Cell Rep. 20, 422–428 (2001). 10.1007/s002990100326 24549450

[b38] Maggert K. A. Reduced rDNA Copy Number Does Not Affect “Competitive” Chromosome Pairing in XYY Males of Drosophila melanogaster. G3: Genes, Genomes, Genetics. 4(3), 497–507 (2014). 24449686 10.1534/g3.113.008730 PMC3962488

[b39] Curie C. . Modular organization and development activity of an Arabidopsis thaliana EF–1 alpha gene promoter. Mol. Genet. Genom. 238, 428–436 (1993). 10.1007/BF00292002 8492811

[b40] Bolle C., Herrmann R. G. & Oelmuller R. Different sequences for 5′–untranslated leaders of nuclear genes for plastid proteins affect the expression of the beta–glucuronidase gene. Plant Mol. Biol. 32, 861–868 (1996). 8980537 10.1007/BF00020483

[b41] Helliwell C. A., Webster C. I. & Gray J. C. Light–regulated expression of the pea plastocyanin gene is mediated by elements within the transcribed region of the gene. The Plant J. 12, 499–506 (1997). 9351238 10.1046/j.1365-313x.1997.00499.x

[b42] De Boer G. J., Testerink C., Pielage G., Nijkamp H. J. & Stuitje A. R. Sequences surrounding the transcription initiation site of the Arabidopsis enoyl–acyl carrier protein reductase gene control seed expression in transgenic tobacco. Plant Mol. Biol. 39, 1197–1207 (1999). 10380806 10.1023/a:1006129924683

[b43] Gutierrez R. A., MacIntosh G. C. & Green P. J. Current perspectives on mRNA stability in plants, multiple levels and mechanisms of control. Trends Plant Sci. 4, 429–438 (1999). 10529824 10.1016/s1360-1385(99)01484-3

[b44] Gutierrez R. A., Ewing R. M., Cherry J. M. & Green P. J. Identification of unstable transcripts in Arabidopsis by cDNA microarray analysis, rapid decay is associated with a group of touch– and specific clock–controlled genes. Proc. Natl. Acad. Sci. USA 99, 11513–11518 (2002). 12167669 10.1073/pnas.152204099 PMC123287

[b45] Syagailo Y. . Structural and functional characterization of the human PAX7 5′–flanking regulator region. Gene. 294, 294–259 (2002). 10.1016/s0378-1119(02)00798-9 12234688

[b46] He X., Futterer J. & Hohn T. Contribution of downstream promoter elements to transcriptional regulation of the rice tungro bacilliform virus promoter. Nucleic Acids Res. 302, 497–506 (2002). 11788712 10.1093/nar/30.2.497 PMC99825

[b47] De Amicis F., Patti T. & Marchetti S. Improvement of the pBI121 plant expression vector by leader replacement with a sequence combining a poly CAA and a CT motif. Transgenic Res. 16, 731–738 (2007). 17237982 10.1007/s11248-006-9063-x

[b48] Bovy A., de Vrieze G., Lugones L., van Horssen P. & van den Berg C. Iron–dependent stability of the ferredoxin I transcripts from the cyanobacterial strains Synechococcus species PCC 7942 and Anabaena species PCC 7937. Mol. Microbiol. 7, 429–439 (1993). 8459769 10.1111/j.1365-2958.1993.tb01134.x

[b49] Dickey L. F., Petracek M. E., Nguyen T. T., Hansen E. R. & Thompson W. F. Light regulation of Fed–1 mRNA requires an element in the 5′ untranslated region and correlates with differential polyribosome association. Plant Cell 10, 475–484 (1998). 9501119 10.1105/tpc.10.3.475 PMC143995

[b50] Kozak M. Structural feature in eukaryotic mRNAs that modulate the initiation of translation. J. Biol. Chem. 266, 1986–1970 (1991a). 1939050

[b51] Gallie D. R. Posttranscriptional regulation of gene expression in plants. Annu. Rev. Plant Physiol. And Plant Mol. Biol. 44, 77–105 (1993).

[b52] Santi L., Wang Y., Stile M. R., Berendzen K. & Wanke D. The GA octodinucleotide repeat binding factor BBR participates in the transcriptional regulation of the homeobox gene *Bkn3*. The Plant J. 34, 813–826 (2003). 12795701 10.1046/j.1365-313x.2003.01767.x

[b53] Meister R. J. . Definition and interactions of a positive regulatory element of the Arabidopsis INNER NO OUTER promoter. The Plant J. 37, 426–438 (2004). 14731261 10.1046/j.1365-313x.2003.01971.x

[b54] Berger N., Dubreucq B., Roudier F., Dubos C. & Lepiniec L. Transcriptional regulation of Arabidopsis LEAFY COTYLEDON2 involves RLE, a *cis*-element that regulates trimethylation of histone H3 at lysine-27. Plant Cell. 23, 4065–4078 (2011). 22080598 10.1105/tpc.111.087866 PMC3246333

[b55] Monfared M. M. . Overlapping and antagonistic activities of BASIC PENTACYSTEINE genes affect a range of developmental processes in Arabidopsis. The Plant J. 66, 1020–1031 (2011). 21435046 10.1111/j.1365-313X.2011.04562.x

[b56] Simonini S. . BASIC PENTACYSTEINE proteins mediate MADS domain complex binding to the DNA for tissue-specific expression of target genes in Arabidopsis. Plant Cell. 24, 4163–4172 (2012). 23054472 10.1105/tpc.112.103952 PMC3517243

[b57] Simonini S. & Kater M. M. Class I BASIC PENTACYSTEINE factors regulate HOMEOBOX genes involved in meristem size maintenance. J. Exp. Bot. 656, 1455–1465 (2014). 24482368 10.1093/jxb/eru003 PMC3967085

[b58] Verma M., Ghangal R., Sharma R., Sinha A. K. & Jain M. Transcriptome Analysis of *Catharanthus roseus* for Gene Discovery and Expression Profiling. Plosone org/10.1371/journal.pone (2014). 10.1371/journal.pone.0103583 PMC4114786 25072156

[b59] Kashi Y., King D. & Soller M. Simple sequence repeats as a source of quantitative genetic variation. Trends Genet. 13, 74–78 (1997). 9055609 10.1016/s0168-9525(97)01008-1

[b60] Trifonov E. N. Tuning function of tandemly repeating sequences: a molecular device for fast adaptation. Pp. 1–24 In Wasser S. P. ed. Evolutionary theory and processes: modern horizons, papers in honor of Eviatar Nevo Kluwer Academic Publishers. Amsterdam, The Netherlands (2003).

[b61] Ma L. & Chung W. K. Quantitative Analysis of Copy Number Variants Based on Real Time Light Cycler PCR. Curr Protoc Hum Genet., 10.1002/0471142905 (2015). PMC3949243 24510682

[b62] Sambrook J., Fritsch E. F. & Maniatis Y. Molecular cloning, a laboratory manual 2nd edn Cold Spring Harbor Laboratory Press, Cold Spring Harbor, N.Y. (1989).

[b63] Kumar S. & Bhatia S. Isolation of *Catharanthus roseus* L. G. Don Nuclei and Measurement of Rate of Tryptophan decarboxylase Gene Transcription Using Nuclear Run-On Transcription Assay. Plos One 105, e0127892 (2015). 26024519 10.1371/journal.pone.0127892 PMC4449189

[b64] Juntawong P., Girke T., Bazin J. & Bailey-Serres J. Translational dynamics revealed by genome-wide profiling of ribosome footprints in Arabidopsis. Proc. Natl. Acad. Sci. USA 11(11), E203–E212 (2014). 24367078 10.1073/pnas.1317811111 PMC3890782

