# Supplementary material for: A polymorphic (GA/CT)n- SSR influences promoter activity of Tryptophan decarboxylase gene in Catharanthus roseus L. Don
Source: Sci Rep. 2016 Sep 13;6:33280. doi: 10.1038/srep33280 (PMC5020687; doi:10.1038/srep33280)

Supplementary Information:

A polymorphic (GA/CT)*n*- SSR influences promoter activity of *Tryptophan decarboxylase* gene in *Catharanthus roseus* L. Don.

Santosh Kumar and Sabhyata Bhatia.

Supplementary Figure Legends

Supplementary Figure 1. Northern blot expression analysis of *Tdc* gene in the two *C. roseus* accessions, Kew1 and Prabal having $(CT)_8$ and $(CT)_{21}$ motifs respectively in the 5' UTR of *Tdc* gene. 10µg of total RNA was probed with cDNA. Ribosomal RNA was used as loading control.

Supplementary Figure 2. Nuclear run-on transcription to measure rate of *Tdc* gene transcription. PCR amplified DNA of *Tdc* (target gene), *Rps9* and SAND (positive control) and MATE (negative control) was blotted on to the nitrocellulose membrane and hybridized with the nascent UTP labeled run-on transcripts from Kew1 and Prabal.

Supplementary Figure 1.

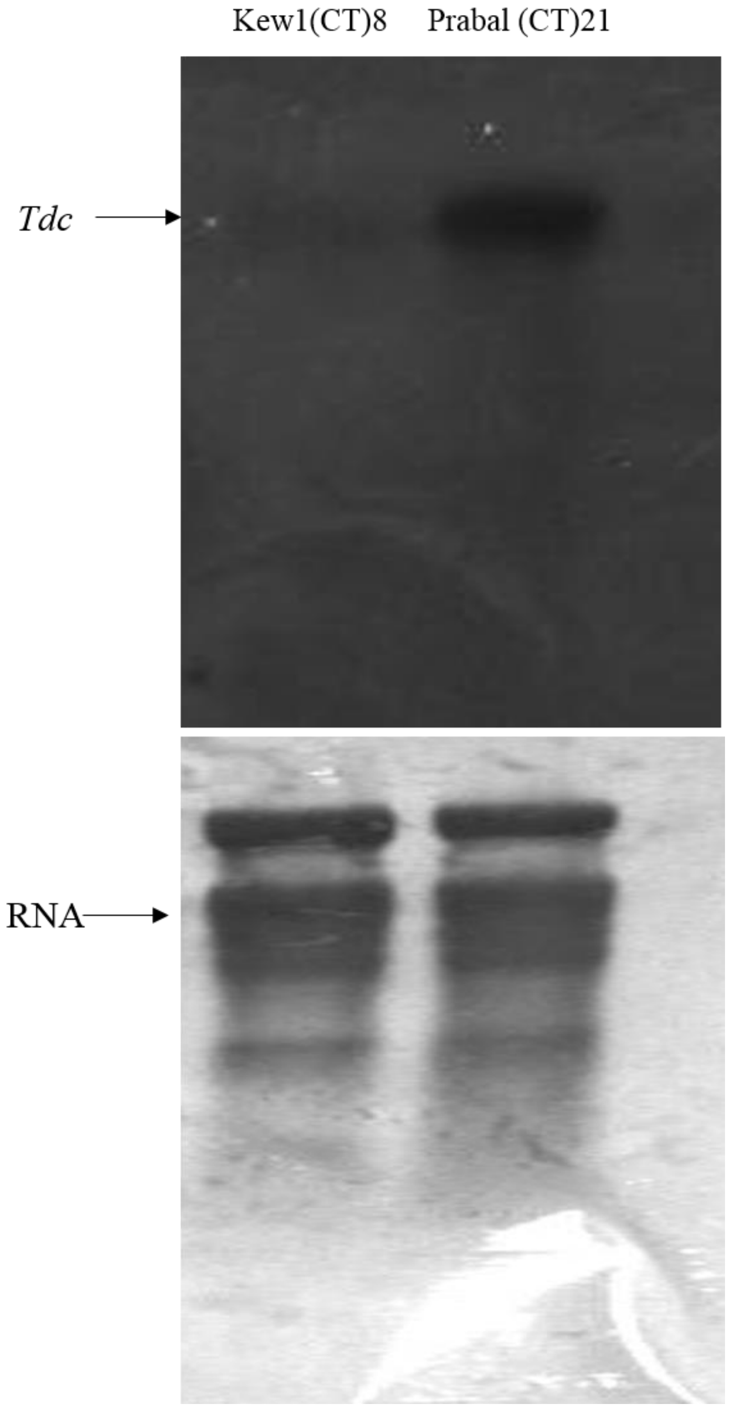

Supplementary Figure 2.

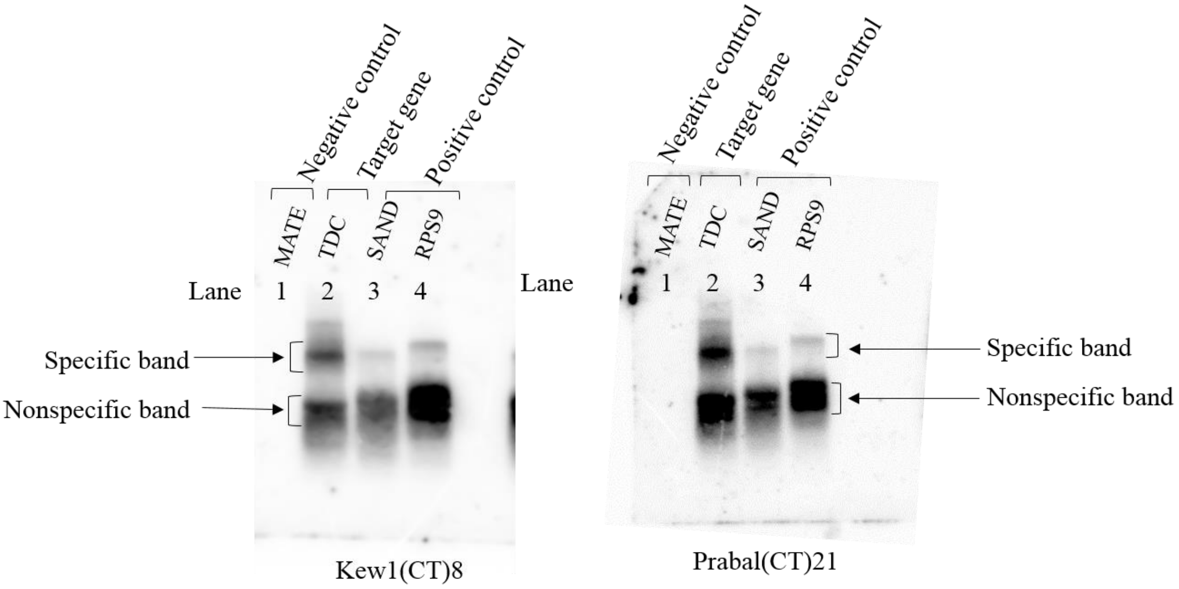

Supplement: Supplementary Information [file srep33280-s1.pdf]
